# Unusual Freshwater-Related Infections Caused by Haematospirillum jordaniae

**DOI:** 10.7759/cureus.25480

**Published:** 2022-05-30

**Authors:** Zachary A Creech, Gia Thinh D Truong, Dorothy X Kenny, Dua Noor Butt, Changzhao Li, Stephen Cavalieri, Rima El-Herte

**Affiliations:** 1 Internal Medicine, Creighton University School of Medicine, Omaha, USA; 2 Internal Medicine, Creighton University School of Medicine, Phoenix, USA; 3 Infectious Disease, Creighton University School of Medicine, Omaha, USA; 4 Pathology, Creighton University School of Medicine, Omaha, USA

**Keywords:** haematospirillum jordaniae, infectious disease, freshwater, cellulitis, bacteremia

## Abstract

Freshwater-related infections can be caused by a broad range of pathogens, potentially leading to skin and soft tissue, pulmonary, gastrointestinal, or even systemic diseases. *Haematospirillum jordaniae *(*H. jordaniae*),a gram-negative, aerobic organism previously regarded solely as an environmental microbe, has been classified as a pathogen capable of causing human infection in the United States. There has been only one other case reported in the literature of *H. jordaniae *infection, and little is known about the pathogenesis.The presentation and progression of clinical symptoms in our cases indicate freshwater cutaneous injury as the most likely route of *H. jordaniae* infection. We present two cases of *H. jordaniae* infection in elderly males. Both patients had freshwater exposure and skin injury resulting in sepsis, cellulitis at the site of injury, and bacteremia. Additionally, one patient presented with an acute deep venous thrombosis. The diagnosis of *H. jordaniae* was confirmed using Sanger sequencing 16s ribosomal RNA data. Antimicrobial therapy included piperacillin-tazobactam, ceftazidime, and levofloxacin. Both patients recovered successfully. While clinical cases and literature involving the newly classified human pathogen* H. jordaniae* are still rare, it is crucial to recognize the potential emergence of environmental organisms, previously believed to be harmless, as human pathogens. In cases of bacteremia and cellulitis with recent freshwater exposure and injury, *H. jordaniae* infection should be considered as part of the differential diagnosis.

## Introduction

Freshwater-associated infections come in many forms, including skin and soft tissue, pulmonary, gastrointestinal, nervous system, and systemic infections. The pathogens involved can vary widely. The common causative agents in skin and soft tissue infections include gram-positive cocci (*Streptococcus* species and *Staphylococcus* *aureus*), gram-negative bacilli (*Pseudomonas aeruginosa* and *Aeromonas hydrophila*), and atypical mycobacteria (*Mycobacterium* *marinum* and *Mycobacterium ulcerans*) [1]. Infection with less common agents such as *Edwardsiella* and *Plesiomonas* or polymicrobial infection can also occur [2,3]. Of these, streptococci and staphylococci are the most common. Empiric antibiotic treatment should include coverage for these organisms and for *Aeromonas* [4]. Fluoroquinolones with a first-generation cephalosporin or clindamycin are typically recommended [5]. If *Mycobacterium marinum* infection is suspected and confirmed, treatment should include clarithromycin or trimethoprim-sulfamethoxazole with rifampin and ethambutol [6]. Definitive therapy requires identification of the pathogen and antibiotic susceptibility testing [7].

*Haematospirillum jordaniae *(*H. jordaniae*), previously regarded solely as an environmental microbe, was recently isolated from human blood cultures in the United States. Hence, the Centers for Disease Control and Prevention (CDC) changed the bacterium’s designation [8]. To our knowledge, there has been only one other report of *H. jordaniae* causing human infection [8]. Here, we present two cases of bacteremia with cellulitis caused by *H. jordaniae *following freshwater exposure.

## Case presentation

Case 1

A 65-year-old Caucasian male presented to the emergency room for generalized weakness, fevers, chills, fatigue, rapidly progressing erythema, edema, and tenderness of his left leg for seven days duration. The patient reported swimming in a local river one week prior and suffering breaks in the skin on his left leg. The patient presented to his primary care doctor five days after the initial incidence and was given oral cephalexin without significant improvement. His past medical history was relevant for chronic lymphocytic leukemia, hypertension, and hyperlipidemia. He was on ibrutinib, atorvastatin, cetirizine, intranasal fluticasone, and polyethylene glycol. His past family history was significant for coronary artery disease. He had a prior cholecystectomy. The patient reported an allergy to sulfonamide antibiotics. He admitted current alcohol use and denied any smoking or illicit drug use. The patient denied any other symptoms.

**Figure 1:**
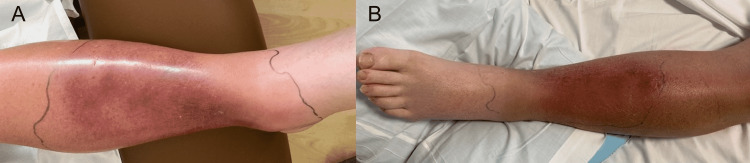
The leg of patient 1 at admission Images show left leg examination upon admission (A) and after three days (B).

On initial evaluation, the patient was alert, oriented, and cooperative. His temperature was 35.5°C, heart rate was 61 beats/minute, respiratory rate was 16 breaths/minute, blood pressure was 130/67 mmHg, and oxygen saturation was 97% on room air. Physical examination was significant for anicteric sclera and well-injected conjunctiva with no conjunctival hemorrhages or suffusion. The patient had adequate dental hygiene without oral ulceration, ear discharge, or pharyngeal erythema. Additionally, the patient presented with normal jugular venous distention, midline trachea with unenlarged thyroid, and no palpable cervical or periauricular lymphadenopathy. His lungs were clear to auscultation, and breath sounds were equal bilaterally. There were no wheezes, crackles, or rhonchi. The chest rise was symmetric, and there were no visible chest wall deformities. His heart examination showed regular S1 and S2 heart sounds with no murmurs, rubs, or gallops. His abdomen was without distention, tenderness, or hepatosplenomegaly. Bowel sounds were normal without palpable inguinal lymph nodes. The patient was well-nourished and non-cachexic. No joints were deformed, erythematous, warm, swollen, or tender. His left leg was violaceous red, warm, and tender, with significant pedal edema (Figure 1). No other rashes were present. He had no wounds, vesicles, Janeway lesions, or Osler nodules. His neurological examination was completely normal and negative for additional findings. His laboratory results at the time of admission are shown in Table 1.

**Table 1:** Laboratory values of patient 1 at the time of admission

Laboratory value (units)	Patient 1	Normal reference values
White blood cell count (WBCs/μL)	13,700	4,500-11,000
Neutrophil count (neutrophils/μL)	10,800	1,500-8,000
Albumin (g/dL)	3	3.5-5
Calcium (mg/dL)	8.3	8.5-10.5
AST (U/L)	14	10-45
ALT (U/L)	21	9-44
Anion gap (mmol/L)	8	<20
Blood urea nitrogen (mg/dL)	16	7-18
Creatinine (mg/dL)	1.09	0.6-1.2
Sodium (mmol/L)	140	137-145
Potassium (mmol/L)	4.1	3.7-5.1
Chloride (mmol/L)	110	96-110
Platelets (platelets/μL)	155,000	140-44,000
Serum glucose (mg/dL)	104	70-100
Hemoglobin (g/dL)	14.5	13.5-17.5
Calculated GFR MDRD non-African American (mL/minute/1.73 m^2^)	71	≥90

**Figure 2:**
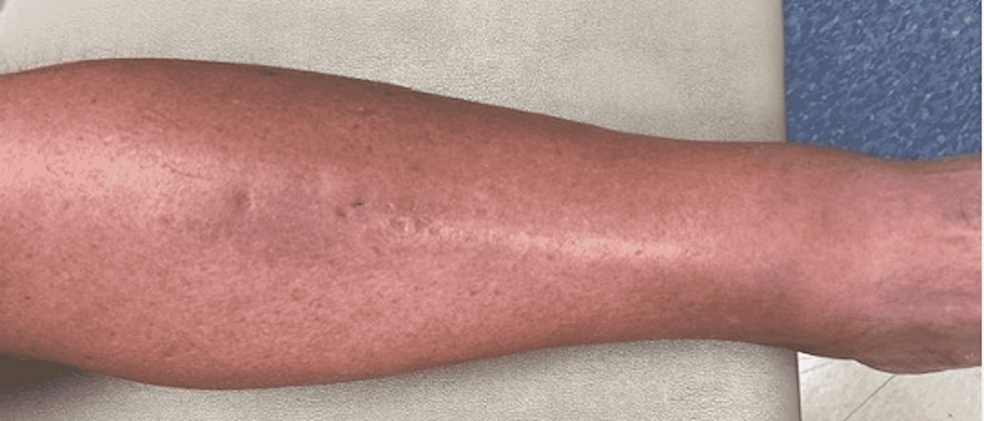
Treatment progression of the first patient The image depicts the first patient’s left leg after 26 days of treatment.

Due to sepsis and cellulitis of the left leg, blood cultures were obtained, and he was started on vancomycin and piperacillin-tazobactam. Infectious disease consultation was obtained, and antimicrobial therapy was changed to vancomycin and ceftazidime. An ultrasound Doppler showed acute deep vein thrombosis of the calf muscle vein on the left side. The blood cultures grew gram-negative rods after five days of incubation, and vancomycin was replaced by doxycycline due to suspicion of tularemia. The patient significantly improved with this treatment regimen, and the erythema and tenderness markedly improved after five days of treatment. Repeat blood cultures were negative for growth. The patient requested to be discharged and continued treatment with intravenous ceftazidime at home, awaiting final blood culture results. Doxycycline was discontinued after discharge. The result of the final blood culture identified the pathogen as *H. jordaniae*. Follow-up at 26 days showed complete resolution of the erythema, warmth, and tenderness with residual dependent pitting edema (Figure 2). At that time, ceftazidime was stopped.

Case 2

A 71-year-old Caucasian male was admitted to the emergency room for erythema, edema, warmth, and tenderness of his left leg. He was swimming in a freshwater lake three days prior to admission and sustained a laceration of the same leg while swimming. The patient reported rapidly progressive worsening left leg pain, erythema, warmth, and swelling. All other reviews of systems were negative.

His past medical history was relevant for hypertension, chronic venous insufficiency, and diabetes mellitus type 2. His past surgical history was significant for remote tonsillectomy. He reported no significantly relevant family history. The patient’s medications included amlodipine, atorvastatin, calcium carbonate, dulaglutide, glimepiride, losartan, hydrochlorothiazide, metformin, and tamsulosin. He had no reported allergies. The patient stated that he was a former smoker and drank alcohol occasionally.

Upon presentation in the emergency room, the patient had a temperature of 39.2°C, heart rate of 116 beats/minute, respiratory rate of 18 breaths/minute, blood pressure of 137/85 mmHg, and oxygen saturation of 95% on room air. He was alert, oriented, and cooperative. An examination of the left leg was significant for erythema and induration, and a break in the skin was noted. Distal pulses and sensations of the lower limbs were both intact. The remainder of the examination was unremarkable. His laboratory results at admission are shown in Table 2.

**Table 2:** Laboratory values of patient 2 at admission

Laboratory value (units)	Patient 2	Normal reference values
White blood cell count (WBCs/μL)	20,800	4,500-11,000
Neutrophil count (neutrophils/μL)	17,900	1,500-8,000
Calcium (mg/dL)	9.1	8.5-10.5
Anion gap (mmol/L)	9	<20
Blood urea nitrogen (mg/dL)	30	7-18
Creatinine (mg/dL)	1.48	0.6-1.2
Sodium (mmol/L)	139	137-145
Potassium (mmol/L)	3.8	3.7-5.1
Chloride (mmol/L)	108	96-110
Platelets (platelets/μL)	192,000	140-44,000
Serum glucose (mg/dL)	191	70-100
Hemoglobin (g/dL)	14.3	13.5-17.5
Calculated GFR MDRD non-African American (mL/minute/1.73 m^2^)	47	≥90

The patient was promptly started on intravenous clindamycin, which was replaced with cefazolin after a blood culture revealed methicillin-sensitive *Staphylococcus aureus* (MSSA). On day 2, blood cultures remained negative. Due to persistent fevers and lack of improvement, an infectious disease consultation was obtained. On day 3 of admission, a gram-negative rod grew from the blood cultures. Cefazolin was therefore switched to piperacillin-tazobactam, and the patient showed rapid clinical improvement. The gram-negative rods later were identified as *Haematospirillum jordaniae*. The patient was discharged on levofloxacin to complete 10 days of treatment. The patient recovered successfully and returned for follow-up after two months.

Methods of identification of the bacteria

**Figure 3:**
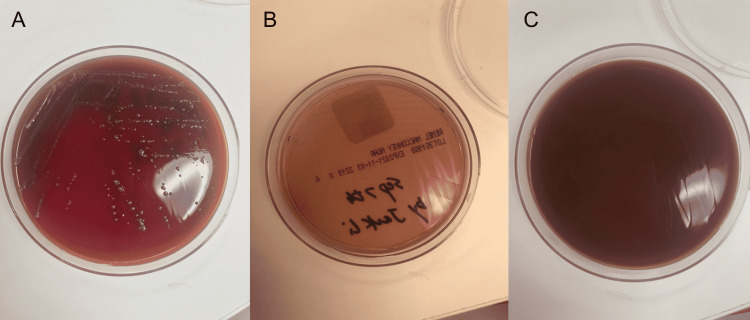
Growth patterns in variable media Pictures show bacteria growth on blood agar (A), but not on MacConkey (B) and chocolate agar (C).

**Figure 4:**
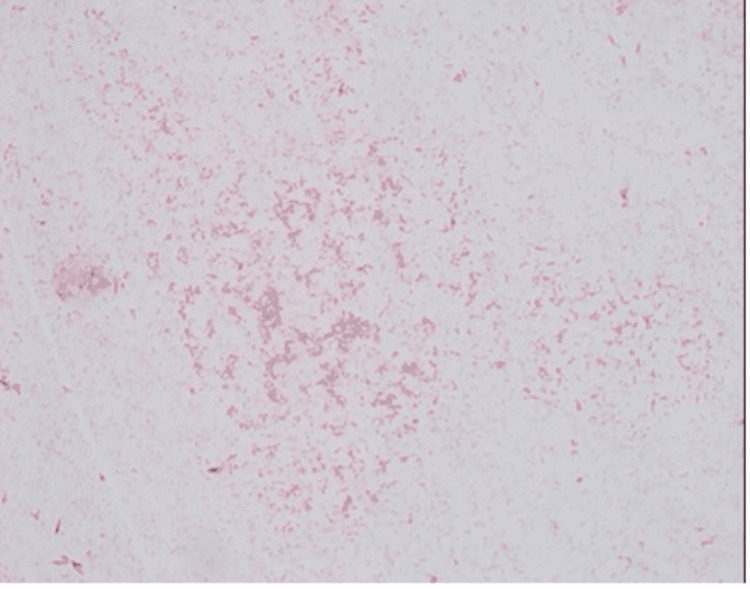
Gram stain showing abundant gram-negative rods The staining depicts faintly stained bacteria in the background.

**Figure 5:**
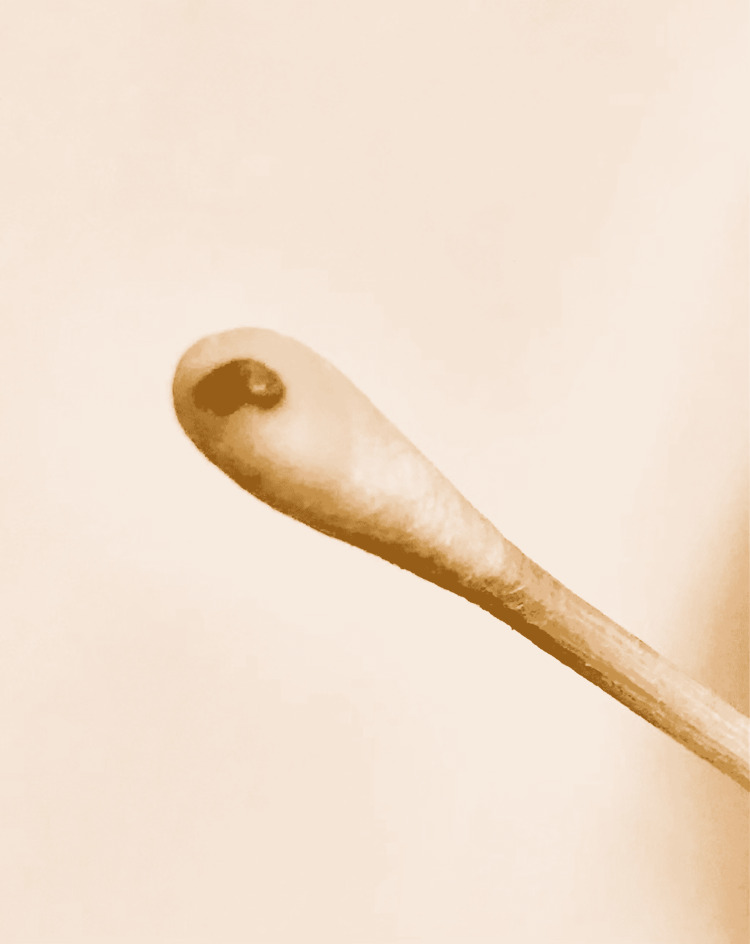
Oxidase test results The blue color indicates a positive oxidase biochemical reaction.

Blood cultures were performed using the BACT/ALERT® Virtuo® system (bioMeriéux, Marcy-l'Étoile, France) with FA Plus (aerobic) and FN Plus (anaerobic) bottles (bioMeriéux). In case 1, the aerobic (FA Plus) bottle became positive after five days of incubation, and gram-negative bacilli were observed. In case 2, the aerobic (FA Plus) bottle became positive after four days of incubation, and gram-negative coccobacilli were observed. Subculture was performed using sheep blood agar, chocolate agar, and MacConkey agar and incubated at 37°C with 5% CO2. Distinct formation of colonies was noted starting from day 5 of inoculation on blood agar only. There was no growth identified on MacConkey or chocolate agars on day 5 and beyond (Figure 3). The colonies were variable in size and demonstrated a gray-white color (Figure 3). Gram stain revealed gram-negative bacilli with variable staining intensity (Figure 4). The oxidase test was done, which was positive (Figure 5).

**Figure 6:**
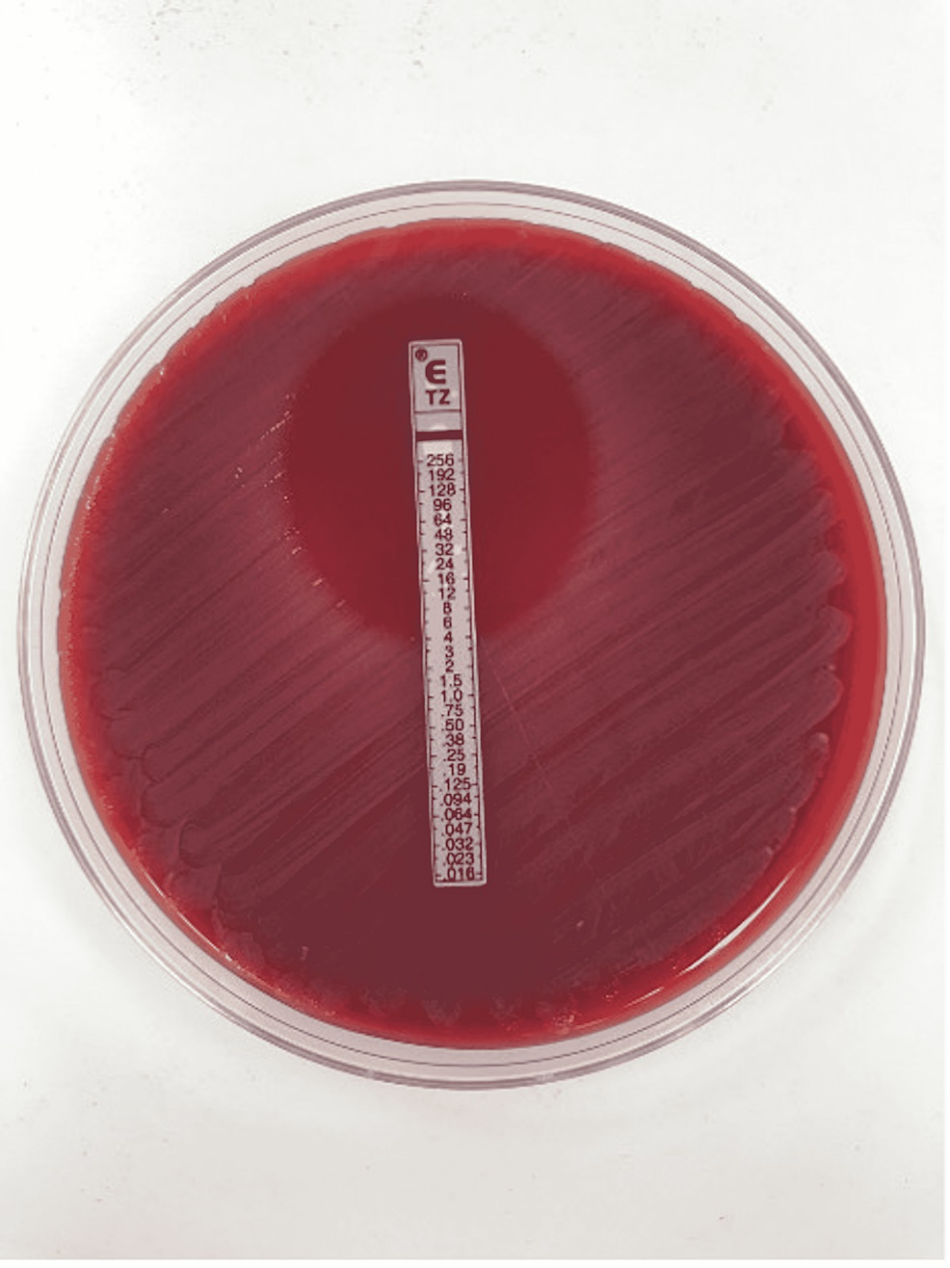
Etest results Ceftazidime Etest shows an MIC of 4 μg/mL.

To further determine the species, Sanger sequencing of bacterial 16s rRNA was performed by the molecular laboratory of Catholic Health Initiatives (CHI) Creighton University Medical Center - Bergan Mercy. The 16s rRNA of the organisms from both cases were matched to *Haematospirillum jordaniae* in the public library available at the National Institute of Health [9]. Antimicrobial sensitivity to ceftazidime was assessed using the Epsilometer test (Etest) (bioMeriéux). A minimal inhibitory concentration (MIC) of 4 µg/mL was observed (Figure 6).

## Discussion

*Haematospirillum jordaniae* is a recently identified bacterial genus and species recognized as a pathogen by the CDC in 2016 [8,10]. *Haematospirillum jordaniae* is a gram-negative, aerobic, motile, spiral-shaped organism with a unique 16s ribosomal RNA sequence in the *Rhodospirillaceae* family [8,10]. *Haematospirillum jordaniae* can be cultured on Heart Infusion Agar supplemented with 5% rabbit blood, producing circular colonies after incubation for 48 hours at a temperature of 35°C [10]. In addition, *H. jordaniae* is positive for hydrogen sulfide production, along with esterase, alkaline phosphatase, and acid phosphatase using the API ZYM test [10]. The bacterium is negative for utilizing urea, nitrate reduction, and conversion to indole from tryptophan [10]. Symptoms reported to the CDC in early clinical cases were nonspecific and included headache, chills, and fevers, along with edema in the lower extremities [10]. These symptoms resembled cases of tularemia, which is caused by *Francisella tularensis*. Additionally, early isolates were all detected in men, with an average age of 60 [10].

In our cases, the patients reported a freshwater injury to the skin and soft tissues with a rapidly progressing infection. The presentation and progression of clinical symptoms point toward the open wound and subsequent exposure to freshwater as the most likely route of infection. A previous case report in 2018 described a similar clinical presentation with lower extremity cellulitis and bacteremia after freshwater exposure. The pathogen was initially suspected to be *Campylobacter*; however, the organism did not grow in a microaerophilic environment. Further biochemical testing revealed the pathogen to be *H. jordaniae*. The patient was successfully treated with aztreonam and ciprofloxacin [8].

Our first patient was successfully treated with ceftazidime for 26 days. The patient’s antibiotic treatment was extended due to his immunosuppression. The patient experienced a left leg deep venous thrombosis during the admission, but it is uncertain if the infection was the direct cause. Repeat blood cultures were negative after five days of treatment with ceftazidime. Etest revealed a minimal inhibitory concentration of 4 μg/mL to ceftazidime (Figure 3). He showed improvement after five days of treatment and complete resolution of the erythema after seven days.

The second patient’s clinical course was complicated by *H. jordaniae* bacteremia and MSSA in his wound. The patient initially responded poorly to just cefazolin but significantly improved with the addition of piperacillin-tazobactam. The patient’s response to the adjusted treatment regimen suggested that MSSA was the colonizer, while *H. jordaniae* was the likely causative pathogen. He was then successfully treated with levofloxacin for 14 days.

To our knowledge, there is limited available literature regarding *H. jordaniae* infections in humans. *Haematospirillum jordaniae* is typically described as a common environmental microbe [10]. This report presents two new cases of *H. jordaniae* infection in two elderly males leading to bacteremia and cellulitis. In one case, a patient developed an acute deep vein thrombosis in his left leg, but the association with *H. jordaniae* infection is unclear. Treatment options include piperacillin-tazobactam, ceftazidime, and fluoroquinolones. There are currently no MIC interpretative breakpoints established for antimicrobial agents against this organism. The definitive identification of this pathogen is dependent on genetic sequencing.

## Conclusions

While clinical cases involving the newly classified human pathogen *H. jordaniae* are still rare, it is crucial to recognize the potential emergence of environmental organisms previously thought to be harmless as human pathogens. In cases with similar symptoms and freshwater injury, *H. jordaniae* should be on the differential.
